# Laser Structured Dental Zirconium for Soft Tissue Cell Occupation—Importance of Wettability Modulation

**DOI:** 10.3390/ma15030732

**Published:** 2022-01-19

**Authors:** Susanne Staehlke, Philip Oster, Susanne Seemann, Fabian Kruse, Jakob Brief, Barbara Nebe

**Affiliations:** 1 Department of Cell Biology, Rostock University Medical Center, 18057 Rostock, Germany; susanne.seemann@med.uni-rostock.de (S.S.); barbara.nebe@med.uni-rostock.de (B.N.); 2 Pulsar Photonics GmbH, 52134 Herzogenrath, Germany; oster@pulsar-photonics.de (P.O.); kruse@pulsar-photonics.de (F.K.); 3 VITA Zahnfabrik H. Rauter GmbH & Co. KG, 79713 Bad Säckingen, Germany; J.Brief@vitaclinical.com; 4 Department Science and Technology of Life, Light and Matter, University of Rostock, 18059 Rostock, Germany

**Keywords:** zirconium, laser micro-structures, cold atmospheric pressure plasma, water contact angle, scanning electron microscopy, in vitro, human gingival cells, cell morphology, actin cytoskeleton, spreading

## Abstract

Various approaches are being pursued to physico-chemically modify the zirconia neck region of dental implants to improve the integration into the surrounding soft tissue. In this study, polished zirconia discs were laser microstructured with periodic cavities and convex waves. These zirconia samples were additionally activated by argon plasma using the kINPen^®^09. The surface topography was characterized by scanning electron microscopy and the surface wettability by water contact angle. The in vitro study with human gingival fibroblasts (HGF-1) was focused on cell spreading, morphology, and actin cytoskeleton organization within the first 24 h. The laser-induced microstructures were originally hydrophobic (e.g., 60 µm cavities 138.4°), but after argon plasma activation, the surfaces switched to the hydrophilic state (60 µm cavities 13.7°). HGF-1 cells adhered flatly on the polished zirconia. Spreading is hampered on cavity structures, and cells avoid the holes. However, cells on laser-induced waves spread well. Interestingly, argon plasma activation for only 1 min promoted adhesion and spreading of HGF-1 cells even after 2 h cultivation. The cells crawl and grow into the depth of the cavities. Thus, a combination of both laser microstructuring and argon plasma activation of zirconia seems to be optimal for a strong gingival cell attachment.

## 1. Introduction

Dental implants are a widely accepted and valuable treatment option to replace missing teeth. In terms of esthetic aspects, zirconia implants can be regarded as a viable alternative to the well-proven titanium implants [1,2]. For many years, the dental implant neck region was machined or highly polished to create a plaque-free area or to meet hygienic conditions [3,4]. For ceramic implants, this practice was adopted from titanium materials [5]. In preclinical studies with dental implants, similar qualitative soft tissue integration was reported by Thoma et al. for zirconia and titanium materials [6]. However, oral and maxillofacial surgeons assumed the maturation processes of the epithelial and connective tissues around zirconia implants were faster, as described by Roehling et al. [7]. The zirconia material alone seems to be preferable for gingival cells, which is an important aspect as gingival tissues around implants have a barrier function and the soft tissue integration is as important as bone integration [8,9].

The implant neck region should also provide topographical and/or chemical characteristics which facilitate mechanically stable and dense soft tissue attachment to promote cell-mediated tissue integration [10]. The function of cells surrounding implants at the implant–tissue interface can be influenced by modifying the material surface properties [3,4]. The initial quality of spreading on a biomaterial is a decisive factor determining the ensuing cell behavior, for example, proliferation, differentiation, and cell type dependent functions [11]. The surface topography of zirconia can be generated by polishing, sandblasting, etching, or laser treatment [2,12,13]. Laser structuring is suitable for creating defined topographies (dimension and shape) on zirconia, and for optimizing the surface characteristics [13,14]. In previous studies with human osteoblasts, we observed improved cell adhesion and growth on femtosecond laser nanostructured and sinusoidal microstructured titanium alloys, regardless of hydrophilicity of the titanium surface [15]. What kind of topography should the neck region of dental zirconia implants have?

To enable sufficient soft tissue attachment to the neck region of the implants and soft tissue integration, smooth surfaces are considered favorable [5,16]. Pacha-Olivenza et al. [17] recognized that the overall “race for the surface” [18] between primary human gingival fibroblasts and bacteria is disadvantageous for the fibroblasts on the rougher ones (acid-etched, sandblasted/acid-etched) compared with the smoother surfaces (machined, slightly acid-etched). More specifically, increasing surface roughness reduced fibroblast proliferation and increased the absolute bacterial adhesion [17], suggesting that smooth surfaces could be preferable in the soft tissue region.

The implant neck region should also provide chemical characteristics to facilitate soft tissue growth. Therefore, different chemical modifications and physical treatments have been developed to enhance cell acceptance and improve support of dental implants, that is, ultraviolet light and cold physical plasma [3,10,19]. Physical plasma is an ionized gas due to high energy supply consisting of ions, electrons, radicals, ultraviolet photons, and uncharged atoms or molecules. Cold physical plasma can be formed under atmospheric or low pressure. These plasmas are used for decontamination/sterilization in dentistry, wound healing, tissue regeneration in medicine [20], or for targeted modification of surface properties of biomaterials [21]. The experimental application of non-thermal plasma for implant material surface modifications is not quite new, thus titanium [19] and its alloys, polyetheretherketone (PEEK) [19], polycarbonate [22], and also zirconia surfaces [19] were treated with argon, oxygen, Ar/1% oxygen [23] or ammonia plasmas for several minutes to obtain improved cell attachment, migration, and growth.

So far, there are few analyses available that focus on how physico-chemically characteristics of the implant influence the cell behavior after the first contact with fibroblasts have been performed [19,24]. Further knowledge of the responses of gingival fibroblasts to the modified surfaces of zirconia would make it possible to specify the manufacturing steps required in the fabrication of dental implants.

The oral neck region of dental implants must be optimally integrated into the adjacent soft tissues to provide long-term clinical implant survival [4]. To achieve this requirement, we combined the influence of surface topography and chemistry on soft tissue cell attachment in vitro. We investigated how HGF-1 cells behave on defined laser microstructures with either various cavity dimensions (concave topology) or waves (convex topology). Additional zirconia surface functionalization was performed with argon plasma (without oxygen) using the kINPen^®^09 plasma jet [25]. The HGF-1 cell response was investigated in terms of cell morphology, cell shape, spreading and actin organization in an early period, within the first 24 h.

The purpose of our work was to find a laser-induced micro-nanostructure for the ceramic surface neck region suitable for human gingival fibroblast on growth. A combined plasma-chemical modification should provide hints about the importance of additional wettability modulation on the gingival cell response.

## 2. Materials and Methods

### 2.1. Zirconia Samples and Laser Structuring

Yttria-stabilized zirconia discs were used with a diameter of 12 mm and a thickness of 1.5 mm. The bulk material, polished zirconia (**Control**), was further micro-structured with the laser microprocessing system RDX1000 and the software Machine control: Photonic Elements (Pulsar Photonics GmbH, Herzogenrath, Germany). The following geometric micro-patterns were created: (i) concave structures: cavity **10/20** with a depth of 10 µm, pitch of 20 µm; cavity **60/120** with a depth of 60 µm, a pitch of 120 µm; cavity **180/360** with a depth of 180 µm, a pitch of 360 µm; and (ii) convex structures (**Waves**): with a depth of 10–20 µm, a pitch of 30 µm and a width of 20 µm (plateau of 12 µm in width/length). The TruMicro2030 laser worked with a pulse duration of <400 fs at a wavelength of 1030 nm (IR) with a raw beam diameter of ~5 mm. The beam was focused by F-Theta optics with a focus diameter of 16 µm. The ablation file is based on CAD/CAM (Computer-Aided Design and Computer-Aided Manufacturing): To create circles, the template was a circle with corresponding dimensions, for the convex structures according to a Waves pattern. Laser ablation was performed along set path vectors (CAM data) at a feed speed of 1000 mm/s. The laser fluence to create Wave structures was 3.37 J/cm^2^ und thus slightly higher than for cavities with 2.9 J/cm^2^. The software Photonic Elements was used for machine control. The process strategy was scanner-based, that is, the laser beam is moved via the scanning system and positioned on the component (component is stationary).

### 2.2. Wettability of Surfaces

The water contact angle (WCA) and the surface-free energy (SFE) of the substrate/air interface were analyzed using the sessile drop method and the Drop Shape Analyzer DSA25 (Krüss, Hamburg, Germany) [26]. One drop (1 µL) of distilled water or diiodo-methane (Sigma-Aldrich, Munich, Germany) was deposited onto the sample surface. Three to five drops per sample, if procurable, were measured regarding the hydrophilicity of the samples. Drop images were acquired with the digital camera of the DSA25. Wettability values were evaluated with the supplied software (ADVANCE, V.1.7.2.1, Krüss, Hamburg, Germany): for WCA the optimal fit method (ellipse, tangent, circle, height/width manual) according to the curvature of the drop shape was used; SFE, dispersive, and polar components, were calculated according to Owens, Wendt, Rabel und Kaelble (OWRK).

### 2.3. Cold Argon (Ar-) Plasma Activation

To modulate the wettability of the polished and laser structured ceramic, the surfaces were activated with the cold atmospheric pressure plasma jet kINPen^®^09 (Neoplas Tools GmbH, Greifswald, Germany) [25,27]. The argon plasma source includes a quartz capillary with a high-frequency (HF) electrode (diameter of 1 mm). An HF voltage (1.1 MHz/2–6 kV) was applied at this electrode. The gas flow of the feed gas argon (99.99%) was 1.9 slm. The settings at the power supply were 60.0 V and 0.05 A. The plasma flame was clearly visible had a length of 12–14 mm and a width about 1 mm. The temperature at the tip of the plasma flame did not exceed 50 °C and was characterized by Weltmann’s group [27]. For the activation of the surfaces, the plasma jet was guided vertically with the quartz capillary 1 cm above the surface in a meandering manner for 60 s, so that the plasma had immediate contact with the whole interface.

### 2.4. Gingival Cell Culture, Morphology, and Spreading

Cell culture: Gingival fibroblasts are able to produce the extracellular matrix molecules, for example, collagen type I and III, fibronectin as well as proteoglycans (including decorin, biglycan, versican, syndecan, perlecan) for maintenance, wound healing, and regeneration of gingival connective tissues [28,29]. Therefore, we used human gingival fibroblasts as competent representatives (HGF-1, ATCC^®^, CRL-2014™, Manassas, VA, USA), which were cultured in Dulbecco’s modified Eagle’s medium (DMEM; high glucose, GlutaMAX; Thermo Fisher Scientific, Gibco, Paisley, UK) containing 10% fetal bovine serum (FBS Premium, South America origin, 0.2 µm sterile-filtered; PAN Biotech, Aidenbach, Germany) and 1% antibiotic-antimycotic (penicillin, streptomycin, and amphotericin B; Anti-Anti 100×, Thermo Fisher Scientific, Gibco) (complete medium). To detach the cells, HGF-1 cells were treated with trypsin/ethylenediaminetetraacetic acid (0.25% trypsin/0.38% EDTA; Invitrogen, Gibco, Paisley, UK) for 5 min. The trypsinization was stopped by the addition of complete medium, and the cell number was measured by NucleoCounter^®^ NC-3000™ (ChemoMetec A/S, Allerod, Denmark). The appropriate number of cells for the experiments was placed in a meandering pattern on the surface, located in a 24-well plate (Greiner Bio-One, Kremsmünster, Austria), and cultured at 37 °C and 5% CO_2_ up to 72 h.

Cell morphology and spreading: The morphology of HGF-1 gingival fibroblasts was analyzed after 2, 24, or 72 h by field emission scanning electron microscopy (FE-SEM, 5 kV; Merlin VP compact, Carl Zeiss, Oberkochen, Germany). For this purpose, the cells were washed with phosphate buffer solution (PBS, Sigma-Aldrich, St Louis, MO, USA), fixed with 2.5% glutaraldehyde (GA, Merck, Darmstadt, Germany), and dehydrated through an ascending ethanol series (30%, 50%, 75%, 90%, and 100%). The samples were dried in the K850 critical point dryer (Emitech, Taunusstein, Germany), and a final step vaporized with carbon under a vacuum (EM SCD 500, Co. Leica, Bensheim, Germany). To illustrate the cells, a high-efficiency secondary electron detector (HE-SE) was used, and an InlenseDuo detector added to image the structures. Subsequently, the FE-SEM images were analyzed by ImageJ (Version 1.51f, Wayne Rasband, National Institutes of Health, Bethesda, MD, USA). For this purpose, the pixels were first converted into µm using the bar of the FE-SEM images (all with the same magnification). After that, the cells are manually marked and then the area, length and width can be analyzed. The quantification of morphometric data—cell area [µm^2^] and length to width ratio—of 40 cells per surface was performed.

Actin cytoskeleton and vinculin: For actin and vinculin staining, gingiva fibroblasts were cultured for 24 or 72 h, respectively, washed with PBS, fixed with 4% paraformaldehyde (PFA, Sigma Aldrich), and further permeabilized with 0.1% Triton X-100 (Merck KGaA, Darmstadt, Germany) at room temperature (RT) for 10 min. For immunolabeling, the primary antibody vinculin-mouse-anti-human (Sigma Aldrich; 1:50 diluted in PBS) was used at RT for 1 h. The secondary antibody anti-rabbit-IgG-AF488 (Invitrogen AG, Carlsbad, CA, USA; diluted 1:100 in PBS) was added at RT in the dark for an additional 1 h. For actin staining, fibroblasts were incubated afterward with phalloidine tetramethyl-rhodamine (TRITC, 1:15 in PBS, Sigma Aldrich) at RT in the dark for 30 min. In the final step, the samples were embedded with Fluoroshield^TM^ with 4′,6-diamidino-2-phenylindole (DAPI, Sigma-Aldrich).

Hyaluronan (HA): HGF-1 gingival fibroblasts were cultured on a cover slip for 24 h and fixed with 4% PFA at RT for 10 min. Subsequently, the biotinylated HA binding protein (2 mg; Calbiochem, San Diego, CA, USA) was applied for 120 min, followed by incubation with streptavidin-fluorescein isothiocyanate (FITC, 0.5 mg; Becton Dickinson, San Diego, CA, USA) at RT for 30 min in the dark [30]. Afterward, the cells were embedded with DAPI-Fluoroshield^TM^.

### 2.5. Confocal Laser Scanning Microscopy

Cell images were recorded on an inverted confocal laser scanning microscope LSM 780 (Carl Zeiss AG, Oberkochen, Germany). For image acquisition, the ZEISS oil immersion 63× objective (C-Apochromat) and the ZEN 2011 (black version) software (Carl Zeiss AG) were used. The images of micro-structures were displayed as three-dimensional (3D) z-stacks. The processing of the images, insertion of the bar, and overlay of the z-stack, were done using the ZENblue software.

### 2.6. Statistic

Statistical analysis was performed with the software GraphPad PRISM Version 7.02 for Windows (GraphPad Software Inc., La Jolla, CA, USA). Data were presented as mean ± standard error of the mean (s.e.m.), or median ± interquartile range (IQR, for wettability). Data analysis was conducted after normal distribution analysis as follows: Ordinary one-way ANOVA post hoc Bonferroni (unpaired, for wettability) or Friedman test post hoc uncorrected Dunn’s test (paired, for spreading analyses). At the level of * *p* < 0.05, differences were considered statistically significant.

## 3. Results

We aimed to elucidate the combinatory influence of laser microstructures and an optional argon plasma activation of zirconia surfaces on human gingival fibroblast (HGF-1) morphology, spreading and growth. Polished zirconia served as controls for the laser-mediated concave cavities in different dimensions as well as convex waves. We used the argon gas-based cold atmospheric pressure plasma jet kINPen^®^09 to additionally activate the ceramic surfaces and determine the importance of the wettability modulation.

### 3.1. Characterization of Human Gingival Fibroblasts

**Figure 1:**
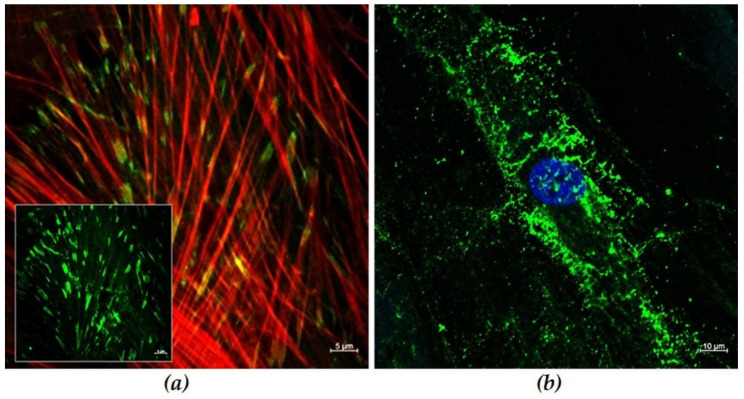
Gingival HGF-1 cellular structures and matrix components: (**a**) The vinculin adaptor proteins (green) co-localize with the fiber ends of the well-organized actin cytoskeleton (red) after 72 h growth (scale bars 5 µm). (**b**) The extracellular matrix molecule glycosaminoglycan hyaluronan is produced by HGF-1 cells in higher amounts (green, nucleus in blue) after 24 h growth (scale bar 10 µm). (LSM 780, Carl Zeiss, Oberkochen, Germany).

The human gingival fibroblast (HGF-1) cells are suitable cell models for in vitro approaches on dental materials destine for soft tissue contact. After 48 h, the cells express a pronounced actin cytoskeleton network with long fibers throughout the cell and well pronounced vinculin contacts as adaptor proteins, important for bridging the integrin receptors with actin fibers (Figure 1). However, due to their origin and fibroblast cell function, HGF-1 cells do not organize tight junctions in cell–cell contacts (not shown). As extracellular matrix producing cells, HGF-1 cells express, i.a., the glycosaminoglycan hyaluronan in higher amount.

### 3.2. Material Surface Characterization

#### 3.2.1. Profiles of Zirconia Samples

**Figure 2:**
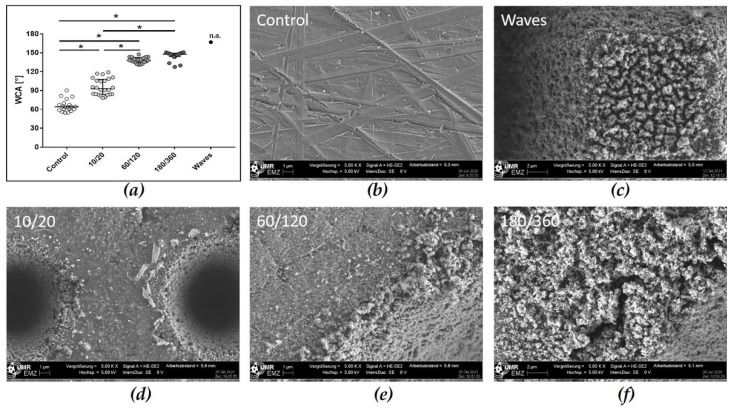
Laser-induced topography of zirconia surfaces and their wettability. (**a**) Water contact angle values of all laser structured samples. Note the superhydrophobic character of cavity surfaces 60/120, 180/360 and Waves. (Scatter dot plot with all measurement points; median ± IQR, * *p* < 0.05, repeated measures ordinary one-way ANOVA and Bonferroni post hoc test). Topographical differences of (**b**) polished zirconia (Control) versus laser-induced (**c**) Waves: 10–20 µm depth, 30 µm pitch; (**d**) 10/20: cavity 10 µm, pitch 20 µm; (**e**) 60/120: cavity 60 µm, pitch 120 µm, and (**f**) 180/360: cavity 180 µm, pitch 360 µm. Note that due to the laser process the zirconia surface is also locally nanostructured with hilly elevations. (FE-SEM Merlin compact, 5000×, 5 kV, Carl Zeiss).

**Figure 3:**
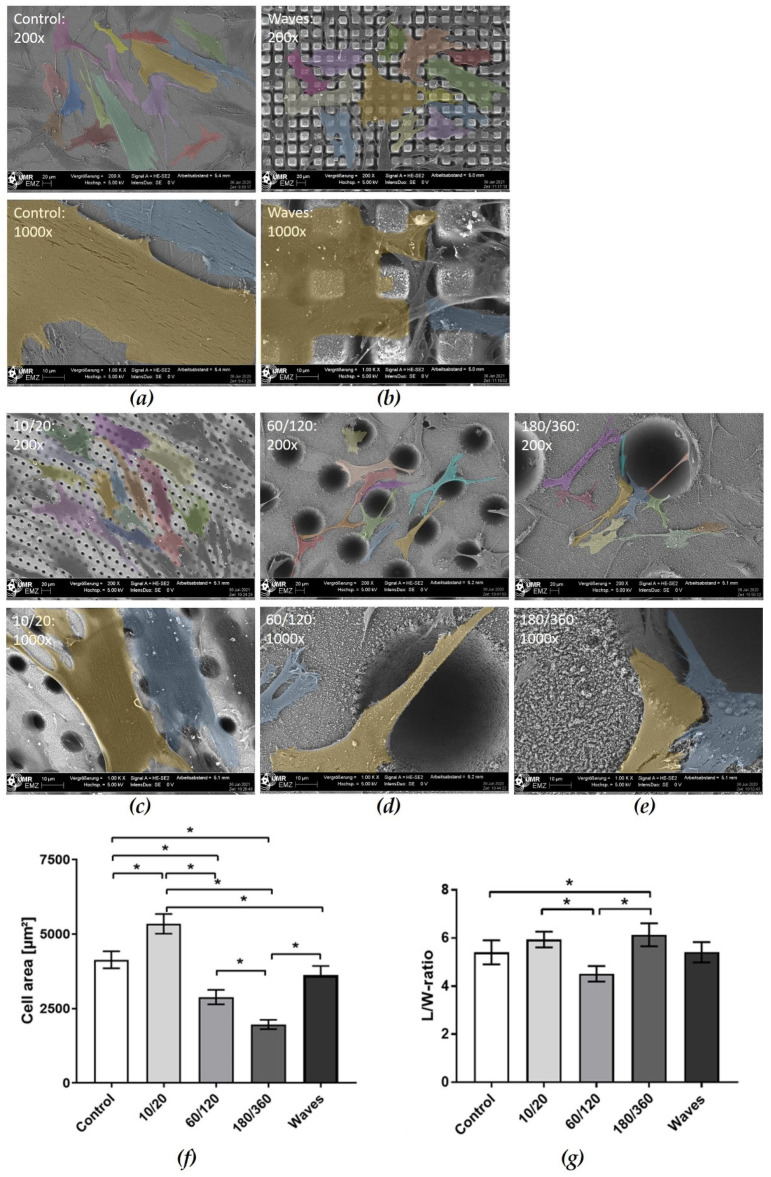
Scanning electron microscopy of HGF-1 gingival cells on various laser structured zirconia surfaces after 24 h growth. Micro-topographical dimensions: (**a**) polished zirconia as control; (**b**) waves: 10–20 µm depth, 30 µm pitch, note that cells grow on and in between the waves; (**c**) 10/20: cavity 10 µm, pitch 20 µm, note that the cells ignore the holes and avoid ingrowth; (**d**) 60/120: cavity 60 µm, pitch 120 µm, note that cells avoid growing into the holes but rather span the holes; (**e**) 180/360: cavity 180 µm, pitch 360 µm, note that cells span the holes but partially migrate also into the depth. (FE-SEM Merlin compact, Carl Zeiss; false colored by PowerPoint software; upper row 200×, bar = 20 µm; lower row 1000×, bar = 10 µm). (**f**) Quantitative data of HGF-1 cell area after 24 h on the laser structures, and (**g**) cell ratio of length to width (L/W). (mean ± s.e.m., Friedman test post hoc uncorrected Dunn’s test; * *p* < 0.05, *n* = 40 cells).

Since the manufacturing processes of the laser structures investigated followed different protocols, we analyzed the resulting topographies by FE-SEM. Regarding the polished zirconia (Control), the FE-SEM images showed that grinding grooves without orientation were detectable (Figure 2). After laser structuring, two types of periodic microstructures can be observed: concave cavities 10/20 holes with a dimension of 10 µm in width and depth, 30 µm pitch; 60/120 holes with a dimension of 60 µm in width and depth, 120 µm pitch; 180/360 holes with a dimension of 180 µm in width and depth, 360 µm pitch, and a sinusoidal, convex microstructure, like pyramid stumps (Waves) with the dimension of 10–20 µm in height and 30 µm pitch (Figure 2 and Figure 3). As the cavities became wider and deeper, the laser had to process the surface more intensively, which was also evident from the nanoporous surface texture of the zirconia (grains) at the marginal areas and in the cavities. On 10/20 cavities, only few nanograins can be detected, but on 180/360 cavities, nano-micro grains are clearly visible (Figure 2f). In contrast, the Waves were characterized by elevations and pits that formed regular pyramidal stumps over the entire surface (Figure 3). The regular structures were always 30 µm apart and had a plateau of approximately 12 µm × 12 µm. The zirconia surfaces also showed a raised nanostructure caused by the laser treatment.

#### 3.2.2. Wetting Properties of Laser Structured Zirconia Samples

The resulting water contact angles (WCA) of the laser-structured surfaces are shown in Figure 2 and Table 1. The polished ceramic reveals a WCA of 65.88° ± 2.15° (Control) and can be classified as hydrophilic [31]. After laser treatment to create cavities of 10/20, 60/120 and 180/360 in dimension, the zirconia surfaces were hydrophobic with WCAs of 95.84° ± 2.67° (10/20), 138.4° ± 0.99° (60/120) and 145.4° ± 1.53° (180/360) (Table 1). The surfaces of the Waves were superhydrophobic, it was not possible to deposit one drop of water on the surface. Thus, we managed to measure a WCA only once (167°). The surface-free energy (SFE) values of the laser structures were comparable, the dispersive part was higher and the polar part was lower in comparison with the Control (Table 1).

To trigger the laser structured samples in a more hydrophilic direction, we activated the samples with argon plasma (Table 1). After plasma activation for only 1 min, the surface displays more hydrophilic characteristics. In particular, the Waves, which were previously superhydrophobic, now switched to a superhydrophilic state. Again, it was not possible to measure the WCA because the drop immediately dispersed on the surface. Due to the hydrophilic states after the Ar-plasma activation, all SFE values were enhanced with a clear increase in their polar parts.

### 3.3. Cellular Behavior

#### 3.3.1. Morphology and Spreading on Laser Structured Zirconia

The different laser structures affected the morphology, spreading and growth of HGF-1 fibroblasts after 24 h (Figure 3). The FE-SEM images showed that HGF-1 cells attached very flatly on polished zirconia (Control). On the laser-induced cavities, cells also spread on the planar areas next to the cavities. The HGF-1 cells were able to span the cavities even over longer distances up to 180 µm (Figure 3c–e). Cells partially avoid growing into the holes of the laser structures with the smaller diameters 10/20 and 60/120, perhaps due to hydrophobic surface characteristics (Figure 3c,d). Increasing the size of the hole up to 180 µm (Figure 3e) does not substantially improve the cell ingrowth. Interestingly, the Wave structures allow for alignment of the gingival fibroblasts in the grooved space (Figure 3b). On the Waves, the cells did not elongate as on the other surfaces, a polygonal cell shape was evident. The HGF-1 fibroblasts were able to become very thin and, above all, to grow into the interstices with their filopodia (Figure 3b).

The quantification of morphometric data—cell area and length to width ratio—was performed from the FE-SEM images using ImageJ (Version 1.51f) (Figure 3f,g). Interestingly, the dimensions of the cavities seem to influence cell spreading. For the smallest cavity 10/20, we could detect significantly increased spreading compared with all other surfaces. Concerning the length to width ratio, we could detect a slight stretching. The spreading decreased significantly on 60/120 cavities compared with the polished zirconia (Control). Here, the lowest L/W ratio could be detected. Interestingly, the spreading on the 180/360 cavity structure was significantly reduced compared with all other surfaces, as indicated by a significantly increased elongation (vs. control and 60/120) especially over the holes (180 µm). The evaluation of the Waves showed a cell area and shape comparable to those of the control. The cell area on Waves was significantly larger than on 180/360 cavity. However, the spreading on Waves could not be detected because a part of the cells disappeared in the grooves beside the pyramid stumps.

#### 3.3.2. Actin Cytoskeleton on Convex Waves

**Figure 4:**
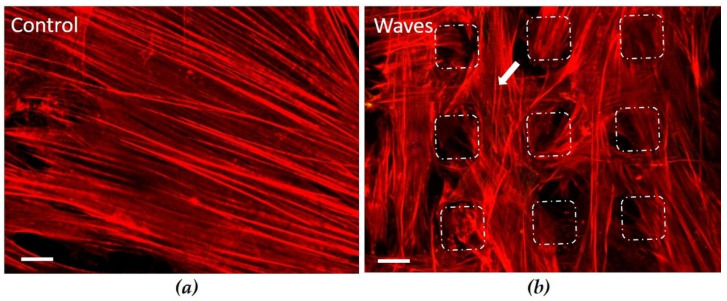
Organization of the actin cytoskeleton in HGF-1 cells after 24 h. (**a**) On polished surfaces (Control), gingival cells formed a well-developed actin cytoskeleton with long filaments which span through the entire cell body. (**b**) On Waves (pyramid stumps: 10–20 µm height, 30 µm pitch), cells grow on the pyramid stumps as well as in the grooves between them (dotted lines, see also Figure 3); long actin filaments are visible in these areas (arrow). (LSM 780, Carl Zeiss; bar = 10 µm, red: actin, white dotted line: plateau of Waves).

Another cell morphological aspect was the organization of the actin cytoskeleton, especially on the Waves (Figure 4). The confocal microscopic images of actin filaments after 24 h of cultivation showed long filaments exactly as on the Controls. No influence of the nano-microtopography on filament length or orientation could be detected. Long actin filaments were detectable both on the plateau of the Waves and between the pyramid-like stumps. On polished zirconia, HGF-1 fibroblasts formed a well-developed actin cytoskeleton with long filaments passing through the entire cell body.

#### 3.3.3. Cell Spreading after Wettability Modulation

**Figure 5:**
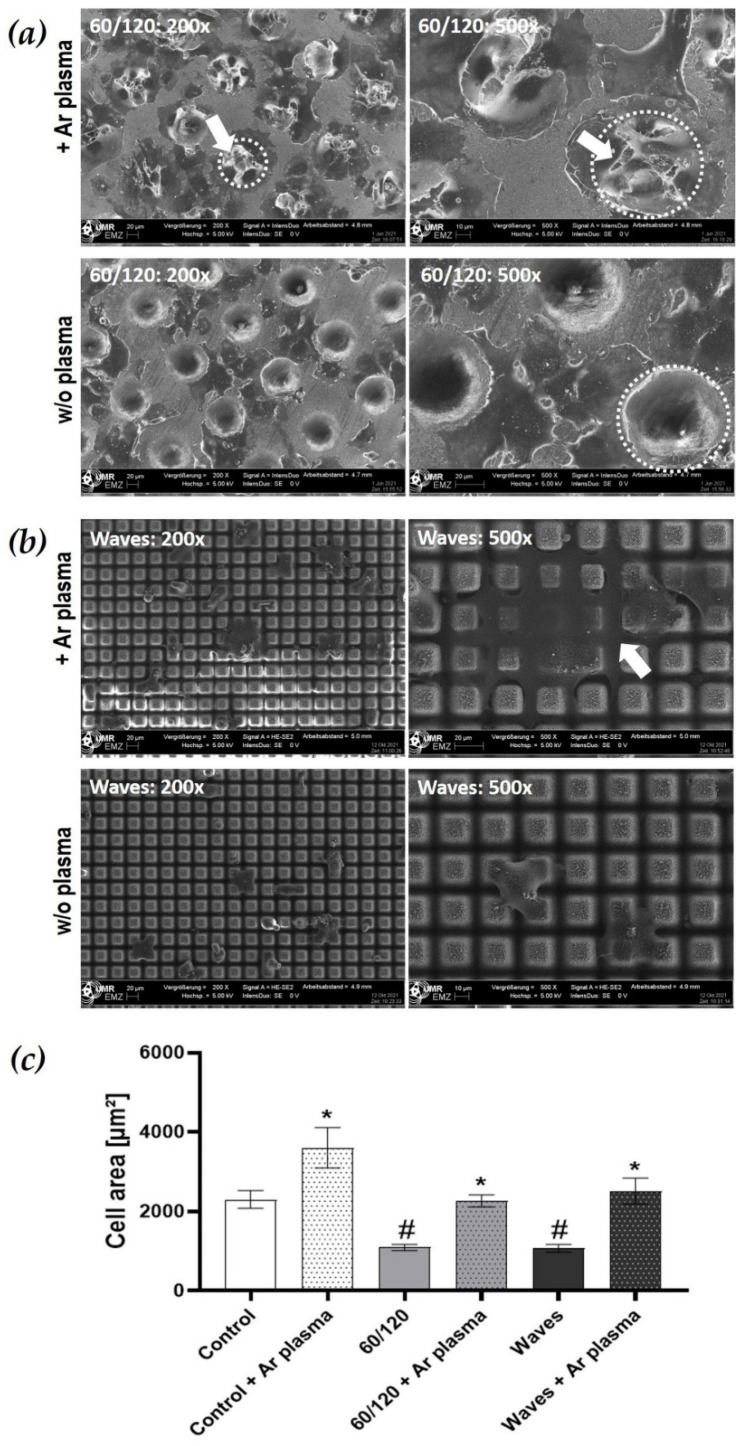
Topography and chemistry—combinatory influence of laser microstructures on zirconia and atmospheric pressure plasma on HGF-1 gingival cell growth. (**a**) Morphology of HGF-1 on 60/120 (cavity 60 µm, pitch 120 µm) after 2 h growth. Note that cells on Ar plasma-activated cavities concentrate their growth more around and in the cavities (arrows), thereby finding a niche. This contrasts with untreated 60/120 surfaces, where cells avoid growing into the depth of the holes (dotted lines around the 60 µm holes). (**b**) Morphology of HGF-1 on Waves (pyramid stumps: 10–20 µm height, 30 µm pitch) after 2 h. Cells on Ar plasma-activated zirconia melt into the valley of the Waves with a large cell area (arrow). This contrasts with untreated Waves, where cells sit on top of the pyramid stumps, remaining small in cell size. (FE-SEM Merlin compact, Carl Zeiss; indicated magnifications 200×, 500×; mode: InlenseDuo). (**c**) Cell spreading after 2 h. Note the significantly increased cell areas due to the additional Ar plasma activation resulting in higher hydrophilicity (see Table 1). (mean ± s.e.m.; * untreated vs. Ar plasma, paired *t*-test, * *p* < 0.05; # compared to control, Friedman test post hoc uncorrected Dunn´s test, # *p* < 0.05; *n* = 40 cells).

Atmospheric pressure argon plasma (Ar plasma) makes the surface more hydrophilic (Table 1). As an illustration, we present the FE-SEM images of HGF-1 cells after 2 h cultivation on the cavity structure 60/120 and on the Waves (Figure 5) after Ar plasma treatment compared with untreated laser structured surfaces (w/o plasma). After plasma treatment with a jet, the surfaces provide better wettability conditions resulting in better cell growth and spreading. Due to Ar plasma activation, a significantly increased cell area could be seen on all surfaces after 2 h compared with zirconia w/o plasma. Only a slightly decreased cell area on all laser structures was detectable compared with the plasma-activated zirconia controls, that is, the chemistry seems to be dominant over the topography and cells are able to spread optimally.

In the FE-SEM images, it could be observed that the cell growth on 60/120 cavity is more concentrated in and around the holes after only a 2 h cell culture following the plasma treatment (Figure 5b). Thus, after plasma activation, the fibroblasts are able to occupy the entire surface, including the niches/holes. This contrasts with the pure, laser-structured ceramic surface. Here, cells grow more between the holes and avoid the ingrowth into the depth of the holes. On the Ar plasma functionalized Wave structures, the HGF-1 cells were huge in the cell area and fuse with the laser structure, especially into the valleys of the Waves. This contrasts with untreated Waves, where the cells after 2 h sit on top of the structure with a small cell area, limited to four pyramid stump-plateaus.

## 4. Discussion

The present study provides insights into how the surface nano-microtopography in combination with plasma-chemistry enables a strong attachment of human gingival HGF-1 fibroblasts on zirconia surfaces. The laser-induced microstructures were originally hydrophobic, but after argon plasma activation, the surfaces switched to the hydrophilic state.

It has been postulated in the literature that physico-chemical surface characteristics such as wettability or roughness influence cell behavior, for example, adhesion, proliferation and differentiation [11,32,33]. It has been found that the cell spreading was improved on smooth surfaces than on moderately rough surfaces [34,35], but roughening the topography leads to a better anchorage of cells following implant integration and final success [4,16,36]. Although the currently available titanium implants are polished on the neck area and are moderately roughened on the endosseous part, the influence of surface topography on implant survival is not yet clearly understood [37]. Other studies have measured the cell behavior of gingiva fibroblasts on zirconia that was either machined [38], etched or sandblasted [35], polished, and/or atmospheric plasma treated [39,40]. Our study introduces a new aspect: defined laser-structured zirconia surfaces.

The laser-structured samples exhibited four different periodic structures that would be applicable to the neck region of zirconia dental implants. We fabricated periodic concave cavities of different widths and depths in the micrometer range—holes with diameters of 10, 60 and 180 µm (see method part) as well as a periodic convex microstructure with rectangular pyramid stumps 10–20 µm in height (Waves). The base material was polished zirconia, which exhibits gridding grooves made by FE-SEM. Enlarged and deeper cavities, as in cavity 180/360, resulted from a more intense laser irradiation, which led to the roughening of the structure and thus to additional nano-microstructures at the edge areas of the holes. The intense laser irradiation results in a significantly increased heat input. Additionally, Rohr et al. [35] were able to show that a graining (comprising tetragonal structures) was detectable by heat treatment of the zirconia.

Another characteristic surface aspect—wettability—is influenced by microstructures and surface roughness, among other factors [3,24,31]. Different studies postulated that surface roughness leads to increased hydrophobic behavior [24]. The cause is the inclusion of air in surface pores, which forms a heterogeneous solid/air surface that impairs spontaneous wetting of the surface (Cassie-Baxter wetting condition) [41]. We could directly demonstrate this with our laser-induced structures 10/20, 60/120, 180/360 concave cavities and the convex Waves. Interestingly, it was not possible to deposit the water droplet on the laser-produced Waves. Thus, the Waves were even superhydrophobic due to the nanostructures on the microstructures (like lotus-leaves). Wang et al. [41] defined the superhydrophobic stages (>150°) and described that the cohesive forces between the water in the needle and the water droplet were stronger than the adhesive forces between the droplet and the ceramic surface.

Functionalization of dental implant surfaces with gas plasmas before clinical use is considered a potential surface modification method for improved implant integration into the tissue [19,24,42,43,44,45]. To investigate the possible biological efficacy of plasma-activated zirconia on samples, the laser-structured surfaces were treated with argon plasma using the kINPen^®^09 for only 1 min. Studies proved that plasma treatment had no effect on the surface topography of the implant materials, but significantly increased the wettability [21,24,46]. In contrast to the laser treatment, plasma activation induced a higher surface free energy (SFE). This rise of SFE with an increase in polar parts was associated with the lower WCA values (hydrophilic characteristic). The effects of plasma activation on WCA and SFE, as well as the negative correlation between both parameters, have already been discussed in the literature [47]. Gentlemen et al. [48] reviewed that the SFE is a relevant feature for cell response. The increase in wettability can be attributed to the removal of carbon contaminants on the surface by the plasma process [42,43,46,49]. Studies could demonstrate that the reduction of WCA due to plasma activation correlated with the initial surface wettability, i.e., as the initial contact angle was higher, the reduction in WCA caused by functionalization was more pronounced [24,49]. This was also observed in our study, the superhydrophobic (>150°) wave structure became superhydrophilic (>0°) after the plasma treatment.

Surmeneva et al. [50] showed that a deposition of nano-hydroxyapatite layers on laser micro-textured titanium resulted in a nano-micro-structured surface topography, which improved surface characteristics such as wettability and SFE.

Human gingival fibroblasts (HGF-1) were used in the present in vitro study to observe the soft tissue response to the different zirconia surfaces more closely. HGF-1 is the main cell type involved in the formation of the mucosal barrier around the implant, which enables successful implant integration [4,38]. Characterization of HGF-1 cells revealed strong actin filaments spanning the entire cells and vinculin contacts at the focal adhesion points after 48 h of cultivation. A pronounced hyaluronan coat was also detected after 24 h of culture, which is important for migration [51], and cell adhesion [30,51].

In their review, Rompen et al. [4] determined that epithelial cells adhered, as well as spread more strongly on metallic surfaces (titanium or gold alloy) than on zirconia surfaces, and modified ceramics promoted cell proliferation to a greater extent than smooth controls. HGF-1 cells on the polished zirconia (Control) spread and exhibited a large cell area that was close to the material surface. On the laser structured cavity 10/20, the cells were able to spread well and spanned the small holes (~10 µm) with their large cell body. The study of Schnell et al. [15] indicated that the size, depth dimension as well as roughness (Sa) of the surface affected osteoblast behavior. Kunzler et al. [16] demonstrated a higher proliferation rate of osteoblasts on rough surfaces, whereas the proliferation of HGF decreased with increasing roughness. We also showed in our study that with increasing pattern dimensions of the cavities, the spreading of HGF-1 was significantly reduced. On cavity 180/360 with the roughest nano-microstructure, spreading was significantly inhibited compared with all other surfaces. Belaud et al. [52] showed in their work that the combination of nanoscale and curved structures stimulated stem cell adhesion and migration. We also observed on the convex microstructure (Waves) only a slight impairment of cell spreading, the morphology and spreading were unaffected and comparable to the polished control. Cells expressed long filopodia that could be observed in the space between the pyramid stumps, that is, in the grooves. Bartold et al. [28] previously observed that fibroblasts expanded during migration and formed filopodia that adhered to the substrate. We could also show this topography-dependent influence of the morphology in previous studies on osteoblasts using defined titanium microstructures [15,53].

Studies suggested that the micro-roughened implant surface has so far served mainly to secure the implant in position, but not to promote initial fibroblast adhesion and proliferation [13,35,54]. A further surface modification—plasma activation of dental surfaces—is already used in practice today, but only for the decontamination of dental implants [21,42]. Concerning the initial spreading of HGF-1 cells on the Ar plasma-activated surfaces, we found significantly increased spreading for all structures compared with untreated materials after 2 h cell culture. Moreover, the FE-SEM images revealed that the cell body of the fibroblasts was closely fused with the surface structure. Most interesting was that the fibroblasts were able to grow into the cavities (60/120) after plasma activation of zirconia, thereby finding a niche. This demonstrates clearly that even inaccessible areas of surfaces can be seeded by cells after plasma functionalization. In other studies, enhanced and improved fibroblast attachment was found on titanium and zirconia surfaces when functionalized with atmospheric plasma [19,55]. Guo et al. [19] compared biomaterial functionalization and found that oxygen plasma supported gingival fibroblast attachment as well as proliferation better than argon plasma activation. Rabel et al. [24] indicated in their proliferation analysis (up to 7 d) no significant differences between HGF on oxygen plasma-treated and untreated control surfaces. Canullo et al. [55] also demonstrated in their study that cleaning with argon plasma accelerated the initial adhesion of L929 fibroblasts, but this effect disappeared after 48 h as a result of saturation.

Discussions of other studies must consider choice of materials, topography, and/or plasma sources. In this respect, Guo et al. [19] found that the effects of plasma functionalization on cell response varied depending on the type of material treated (titanium vs. zirconia).

In this study, the limitation from the cell biology point of view is the usage of only one human cell type as representative of the soft tissue. This should be expanded in further experiments. Ingrowth of cells and the acceptance of the material topography is a precondition for the cell physiology and function. However, in the following experiments it should be observed how HGF-1 cells synthesize extracellular matrix molecules (e.g., collagen I, fibronectin, hyaluronan). This preliminary study was limited to the morphological behavior of HGF-1 gingival cells. Our research raises new questions, and further studies are needed to understand cell behavior at the interface of differently modified zirconia surfaces more precisely. The present results suggest that, in the development of laser structured dental implant surfaces for improved tissue integration, argon plasma activation would be a possible compatible method for advanced modification approaches [24,42]. In particular, the plasma treatment with a plasma jet enables the activation of dental implants directly before insertion in clinical practice.

## 5. Conclusions

Laser-induced nano-micro texturing of zirconia surfaces seems to be a good tool for producing defined topographic patterns to control cell growth. However, micro-rough surfaces caused by the laser process showed decreased surface wettability accompanied by reduced cell spreading. An activation of the laser-structured surface using argon plasma can equalize these effects. The zirconia surfaces switched to the hydrophilic state with a higher SFE and the cell areas significantly increased. In addition, cells could find a niche in the laser-induced holes and the grooves. Within the limits of the present study, it can be concluded that the argon plasma activation of zirconia is a potential method for increasing the wettability of structured zirconia with its defined laser-induced patterns and providing a stimulus for improved HGF-1 cell attachment and ingrowth.

## Figures and Tables

**Table 1:** Surface characteristics of laser structured zirconia surfaces before (w/o plasma) and after argon plasma activation (+Ar plasma). Note the extreme switch from the superhydrophobic to the hydrophilic state of convex Wave structures.

Surface	Method	Control	10/20	60/120	180/360	Waves
**w/o plasma**		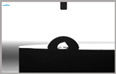	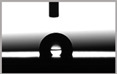	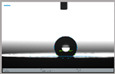	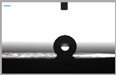	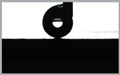
	**WCA [°]**	**65.88 ± 2.15**	**95.84 ± 2.67**	**138.4 ± 0.99**	**145.4 ± 1.53**	**≥150**
	**SFE [mN/m]** dispersive polar	**46.82 ± 4.29** 31.21 ± 1.62 15.61 ± 2.67	**48.12 ± 1.45** 47.34 ± 1.45 0.78 ± 0.89	**54.84 ± 3.09** 45.51 ± 1.53 9.32 ± 1.56	43.13 ± 3.60 33.89 ± 2.52 9.24 ± 1.08	
**+Ar-plasma**		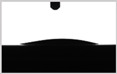	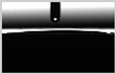	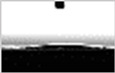	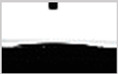	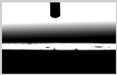
	**WCA [°]**	**17.27 ± 1.35**	**7.34 ± 2.41**	**13.67 ± 1.13**	**10.25 ± 1.12**	**≥0**
	**SFE [mN/m]** dispersive polar	**72.46 ± 6.21** 35.26 ± 3.35 37.20 ± 2.86	**80.61 ± 0.4** 50.56 ± 0.26 30.05 ± 0.13	**77.65 ± 1.75** 46.39 ± 0.71 31.25 ± 1.06	**79.96 ± 1.05** 49.68 ± 0.34 30.28 ± 0.71	

## Data Availability

The datasets generated during and/or analysed during the current study are available from the corresponding author on reasonable request. The dataset is stored on the local UMR server.
